# Homoeopathy a True Science

**Published:** 1892-10

**Authors:** J. H. Allen

**Affiliations:** Logansport, Indiana


### INTERNATIONAL HAHNEMANNIAN ASSOCIATION MEETING OF 1892.

*First Day—Evening Session.*

Tuesday, June 21st, 8 P. M.

(Bureau of Homœopathic Philosophy—Continued.)

### HOMŒOPATHY A TRUE SCIENCE.

J. H. ALLEN, M. D., LOGANSPORT, INDIANA.

Any science, in order to be a true one, should have a philosophical basis, without which it is more or less empiricism and cannot be classed as such.

It requires certain fixed laws that are not only natural, but universal. The atom must be governed by the same law as the molecule, and the molecule as the mass. Even at the close of the eighteenth century, most of the sciences were in an empirical state. Take for instance, Astronomy. All was chaos until Kepler founded our modern science. The best recognized function of the German school at that day was their construction of prophecying almanacs—greedily bought by a credulous people, and quickly believed by the future they pretended to disclose. But Kepler followed out the suggestions of Ptolemy and Cardey, on planetary influences, together with his wonderful mathematical calculations as to planetary changes; but not until a Newton came and gave us that universal law of gravitation could it be classed as a true science. So it is with one of the most beneficial of all sciences to man—medicine. Away back in the almost legendary past, when knowledge was but a feeble taper burning in the dark, came up an Hippocrates as a bright flame lighting up the horizon of that trackless waste where the foot of science had never trod. But under him who said "My work is not weighed or measured," the dark clouds of Mythological medicine began to disappear, with all their superstitions, with all their foolish prayers, sacrifices, and mysterious incantations. This was but the breaking of the dawn. One by one the great lights began to burn as we follow down the ages —forerunners of the great Messiah of medicine that was to come.

In the course of time came that prince of philosophers—Samuel Hahnemann, the founder of a School of Philosophy that is greater than that ever taught in the Porch of Zeno; greater than the Lyceum of Aristotle; greater than the Academy of Plato. Yes, greater for the benefit of mankind, than any other philosophy. His law of cure is the true essence of philosophy, God-given in its principles; true as the laws that govern the motions of spheres.

I have divided Hahnemann's philosophy of medicine into three great fundamental principles:

First—The dynamic origin of disease.

Second—The law of the similars, and their application to the cure of disease.

And the third great principle is the potentiation of drugs.

In the first principle we will place life—its nature, origin, and the relationship it bears to disease.

To define life has been but the feeble attempt of the few. Socrates, the father of philosophy and reasoning, had worked out that great problem of immortality, and the continuance of this life beyond this vale of tears; but he who said, "It must be so, Plato, thou reasonest well," spoke not of that vital principle that vivifies and animates these corporeal casts of clay. Swedenborg says, "Life is that constant efflux from the Almighty." "Life," says the German physicologist, "pre-exists organism. It collects matter or material, and creates organism. It is the priors of all creation. Nature, in all its immensity, is but the outfolding of the absolute." Samuel Hahnemann, though not the first to recognize this life-force as the prior of organized matter, was the first to recognize disease or a diseased condition as but a deranged or disturbed life-force. "Health," he says, "is these forces acting in harmony. Disease is deranged life-force, still retaining its spirit-like origin." Thus he reasoned: "If this be the true philosophy of disease, this material organism depends entirely on this vital principle, and is incapable of sensation, action, or self-preservation without it, it is this vital principle alone that animates it either in health or disease, imparting to it all sensation, and enables it to perform its function." Reasoning from this standpoint, the master mind of Hahnemann began to search for a law of therapeutics, which brings us to the second great fundamental principle of his law of cure, or the law of the similars.

Long before Hahnemann's day others had alluded to this law of the similars as a law of cure. Noted among them was Paracelsus, born about 1490. He, like Hahnemann, was a great chemist; and it is said that he made a study of mineral waters and used them in the cure of disease. His doctrines were confirmed to some extent by his success in curing diseases, or mitigating them, with which other doctors could no nothing. But after expounding his doctrines to his friends they began a series of persecutions against him which drove him from the University, and even from his native home, and from them he suffered more persecutions than did Hahnemann. The question now arises: Did Paracelsus at any time or in any way adopt the law of the similars in his cure of disease or not? The probability is that he did not; but it is possibly true that he had conceived of disease as some phase of life. On the other hand, Did Hahnemann receive any insight from the writing of Paracelsus or others? Some have thought he did. At least his enemies have accused him of having stolen his knowledge from others, and that he is not the first to have discovered the law of the similars. Hahnemann does not say that he was the first to conceive of diseases being cured by their similars. Hypocrates had said "Sometimes diseases are cured by their similars and sometimes by their opposites," but he does say that he was the first to put the law of the similars into practice and conceived it to be a universal law. The similarity of the symptoms that China produced on himself to the Hungarian fever probably formed a nucleus to a train of thought that led him to make this wonderful discovery, together with the analogies taken from the open book of nature, of which Hahnemann was a close student, and to which he refers us all through his *Organon*, helped to strengthen his belief until he had proven enough remedies to put it into practice. Pure experience through the senses, which was the philosophy of the school from which he was about to break loose, was an ignominious failure and would not assist him; but pure experience through the understanding did assist Hahnemann in working out this wonderful law whose motto is the finger-board pointing the way. He did not call it a science of biology or consign it to that sphere. He simply noted and studied the phenomena and gave it to us as a well verified and only scientific law of cure. In paragraph twenty-eight of the *Organon* he says: "I therefore place but slight value upon any attempt at an explanation, but the following holds good as the most probable: The artificial morbid disease is implanted upon the vital power deranged by the natural disease," etc. It would seem that for us to know just how a remedy or these disease-producing agents act is beyond the ken of our senses and probably the understanding, though not out of the sphere of the natural. We can but note the phenomena and appreciate the wonderful workings of the law. In the first place, we note that we are dealing with force agents. "What is force?" "It is an active state of matter," says the scientist, and "What is matter?" Why! force partially at rest or in a placid state. Can we consign the action of the homœopathic remedy to the law of motions? or, in other words, through the law of action and reaction? This is a pure understanding conception, and may be verified in an objective reality by the determination of an experience as contemporaneous or as occurrence of events simultaneously. We can conceive of substance in combinations or in collision, while certain modification must be made in one or the other substance and thus the change be reciprocal, whether the natural disease-producing force forms a combination with the artificial disease-producing force and neutralizing the former so that it is no longer a disease-working agent. Perhaps some negative or positive state, as the case may be, is set up by the conjunction of these two contending forces are questions for scientific thought. However it may be, it matters not much whether we know or not just how these wonderful changes take place in our patients when we have given the right remedy, so long as we walk under the banner of the law, the potent drop will still work its wonders before our eyes.

The last fundamental principle of the great law of cure is the potentiation of drugs. The discovery of the dynamic origin of disease and the law of similars may be considered as among the greatest gifts that could have been bestowed on the human family, but the crowning glory of this beautiful philosophy is his potentiation of drugs. Hahnemann, no doubt, like the most of us when we first began to practice Homœopathy, used tinctures and drugs in a crude state, then gradually began to lessen the dose until finally it reached that point when it was infinitesimal. "When," says Hahnemann, "that point was reached where neither physics, chemistry, or any method known to science could detect any trace of the original drug, still it was a powerful disease-producing agent." To use his own words, it exalted its power; it acted longer, deeper, and gave better results.

Upon this he based the last great principle of his law of therapeutics—that is, disease is of dynamic origin, and is cured by producing an artificial disease similar to the natural one. Then the artificial disease-producing agent must be of a spirit-like nature.

We then must have at our command, in order to fight our battles well with these unseen forces that derange and distune these healthy life-forces, agents that have like qualities, the nature of which is similar. It must be graded and tuned to a similar note or key. Crude and inert matters have not these qualifications until they are potentized. It is a sort of development, arising from a lower to a higher plane in the quality of power. In the crude state it was under the law of chemistry—more mechanical : now it approaches the dynamic or the action of the forces unseen. They are no longer clothed with the material which was but a medium from which these potent forces were liberated. It is the key that unlocks the secret doors and liberates these Prometheus-bound forces from their storehouses of nature, that we may use them in our battles with disease.

The action of the crude drug, and even the lower potencies is like a picture that is roughly sketched. It is only in the first stages of development, and lacks tone and harmony. The colors do not blend nicely ; it needs here and there the skillful touch of a master hand to bring it out. The crude lines must be modified ; the dark shadings toned down with light, when we have before our eyes a perfect picture—a thing of beauty. The action of the higher potencies is more prolonged, and does not require frequent repetition—usually one dose or two suffices to bring about the desired result as far as the action of that remedy is concerned. Its action is more profound, changing even mental and often moral conditions. The very solar centres of life give us demonstrations of their presence. There are none of you but have seen their struggles with that grim monster, Death, for mastery, contending with those malignancies that would seem to scoff at their feeble efforts, and when your patient was sinking, sinking in that downward groove that leads to death, your potencies came out victorious.

In conclusion, let me ask you to study well this Hahnemannian philosophy, the basis of which is found in his *Organon* of medicine, every paragraph of which is a mine of knowledge; every line containing a precept or a principle. Let us study well our materia medica, for it is our sheet-anchor; study well these potent forces that bring back life and health and happiness to our patient and make us feel that the world is better for our having lived in it.

